# Evaluating the Effectiveness of Nonpharmacological Self-Management Interventions for Persistent Pain: Protocol for Single-Case Experimental Designs

**DOI:** 10.2196/79810

**Published:** 2026-06-15

**Authors:** Nancy Sturman, Jane Nikles, Peter Worthy, Shauna Fjaagesund, Rachel A Elphinston, Nicole Andrews, Michele Sterling, Stefan Konigorski

**Affiliations:** 1 General Practice Clinical Unit The University of Queensland Brisbane Australia; 2 School of Engineering and Computer Science The University of Queensland Brisbane, Queensland Australia; 3 Health Hub Doctors Morayfield Morayfield, Queensland, Queensland Australia; 4 RECOVER Injury Research Centre The University of Queensland Brisbane, Queensland Australia; 5 STARS Education and Research Alliance, Surgical Treatment and Rehabilitation Service (STARS) The University of Queensland and Metro North Health Brisbane, Queensland Australia; 6 NHMRC Centre of Research Excellence: Better Health Outcomes for Compensable Injury The University of Queensland Brisbane, Queensland Australia; 7 Tess Cramond Pain and Research Centre The Royal Brisbane and Women's Hospital, Metro North Hospital and Health Service Herston Australia; 8 The Occupational Therapy Department The Royal Brisbane and Women's Hospital, Metro North Hospital and Health Service Herston Australia; 9 Hasso Plattner Institute, Digital Engineering Faculty University of Potsdam Potsdam, Brandenburg Germany; 10 Windreich Department of Artificial Intelligence and Human Health Icahn School of Medicine at Mount Sinai New York, NY United States; 11 Department of Computational Precision Nutrition German Institute of Human Nutrition Nuthetal Germany

**Keywords:** general practice, N-of-1 trials, single-case experimental designs, pain, mobile health, mHealth, self-management

## Abstract

**Background:**

Nonpharmacological self-management interventions are often recommended for general practice patients with persistent pain—a distressing, costly, and heterogeneous condition with variable responses to treatment interventions. Single-case experimental designs (“self-experiments”) evaluate intervention effectiveness at the individual level. StudyU is an open-source digital platform designed to help patients undertake these self-experiments.

**Objective:**

This study aims to investigate the feasibility and acceptability (for patients, practitioners, and other practice staff) of integrating digitally enabled, withdrawal/reversal self-experiments in the general practice care of patients with persistent pain.

**Methods:**

We will recruit 50 patients from a large Australian general practice. Participants will trial a self-selected, self-management intervention (such as physical activity, mindfulness practice, or online self-guided cognitive behavioral therapy) approved by their general practitioner (GP) and use the StudyU app to rate the daily impact of their pain over the 10-week study period. The primary clinical outcome of the self-experiments is pain interference (measured using the modified Brief Pain Inventory), testing for its mean difference between usual routine and intervention conditions. Clinical reports will be generated for the patient and their GP. We will use validated measures of app usability and acceptance, pre-post measures of patient self-efficacy, quality of life, health service use, self-reported health; individual interviews informed by the Normalization Process Theory; and a nested process evaluation to examine the feasibility and acceptability for patients and practice staff of embedding these self-experiments in general practice care.

**Results:**

The research was funded in September 2023, funding and other agreements were completed by December 2024, patient recruitment commenced in January 2025, and publication of results is anticipated by January 2027.

**Conclusions:**

Digitally enabled self-experiments testing nonpharmacological treatment effectiveness may empower patients to self-manage persistent pain and adopt personally effective nonpharmacological interventions in partnership with their GPs and provide a model for integrating other new technologies into the general practice care of patients with other chronic conditions.

**Trial Registration:**

Australia and New Zealand Clinical Trials Registry ACTRN12624000459527; https://tinyurl.com/bdhabjd2

**International Registered Report Identifier (IRRID):**

DERR1-10.2196/79810

## Introduction

### Persistent Pain

Innovative and practical solutions are needed to address the costly and distressing problem of persistent (or chronic) noncancer pain. Living with persistent pain is associated with substantial disability [1] and health care system costs [2]. Persistent pain also presents challenges for general practice, including navigating ongoing compensation and insurance matters, and patient dependence on pharmacological interventions [3]. Although nonpharmacological, self-management, and resilience-building options for managing pain are often recommended, persistent pain is a highly heterogeneous condition with variable individual responses to treatments [4]. Single-case experimental designs (personalized, single-patient self-experiments using patients as their own controls) [5] are well positioned to evaluate effectiveness at the individual level [4], but there is very limited evidence about the feasibility and acceptability of adopting these in general practice.

### Digitally Enabled Self-Experiments: StudyU

The open access StudyU app is a mobile phone app designed for conducting single-case experimental designs [6]. In this study, we use the app to enable patients with persistent pain to evaluate the effectiveness of their choice of a self-management, nonpharmacological intervention. We have piloted the app with 4 community-based consumers with persistent pain. The app is embedded in the StudyU platform [6], which also includes a general-use tool for researchers and health care practitioners to design, monitor, and manage these patient self-experiments.

The StudyU app will generate daily reminders for patients to undertake their chosen activity, and rate pain severity and impact over a 10-week test period. After patients complete their self-experiments, the app will provide visual displays of daily ratings and average baseline vs intervention ratings, using color-coded bar graphs. The research team will also analyze outcomes using *t* tests and Bayesian linear mixed models to compare baseline and intervention conditions, producing clinical reports that are forwarded by a secure messaging system to the patient and their general practitioner (GP). StudyU does not require a user account and does not store any personal identifying data. The anonymized recorded study data will be published after study completion to contribute to an open worldwide data repository, with patient consent, to allow aggregation of single-case experiments testing similar interventions in future open science approaches, and to inform the design of future trials.

### Apps in General Practice

Apps are used in general practice to improve consumer adherence [7], monitor symptoms [8], supplement medical histories [9], and implement management algorithms [10]. More than 50% of Australian GPs in a 2019 survey study recommended mobile health apps to patients at least monthly [11]. However, GPs also perceive barriers to effectively adopting evidence-based apps, including limited awareness of suitable apps, and concerns about time commitment, privacy, safety, and trustworthiness [12]. Australian patients appear less concerned than GPs about privacy and data safety issues and appreciate their physicians recommending evidence-based apps [12], although social and economic disadvantage (particularly low income, education, and employment) and rural location may reduce digital access, affordability, and ability [13,14]. In international literature, workflow adjustments, inadequate reimbursement, and high training effort are substantial barriers for digital health adoption by GPs, whereas interoperability, integration with workflow, continued technical support, improved usability, digital formularies, payment models, and attention to personal and emotional elements facilitate uptake [15,16]. The engagement of both health professionals and patients is essential for successful integration [12].

### Aim

The overall aim of this research is to empower patients with persistent pain and their treating teams to adopt effective self-management activities and discontinue treatments that are ineffective for them personally (even if these are generally recommended).

### Research Question

The following was the research question: Is it feasible, acceptable, and useful to embed digitally enabled self-experiments into a general practice setting to test the effectiveness of nonpharmacological treatments in patients with persistent noncancer pain?

## Methods

### Ethical Considerations

#### Overview

Ethics approval for this study was obtained from the University of Queensland Human Research Ethics Committee (2023/HE000039). Written informed consent was obtained from patients and all interview participants after providing written participant information which included the nature and consequences of the research, and the ability of participants to opt out at any stage. Gift vouchers will be provided to practice nurses to reimburse them for time spent recruiting and assisting patients. Study nurses and GPs will also be reimbursed for attendance at a training session to familiarize them with the study rationale and procedures and for participation in individual interviews at study completion. Gift vouchers will be provided to patients to reimburse them for the initial study visit, survey completion, and poststudy interviews.

#### Data Storage and Data Privacy

Different types of data will be assessed during the study. Questionnaire data from baseline and after 4 weeks, as well as interview data, will be collected and stored locally at the University of Queensland. All data from the single-case experimental designs will be recorded using the StudyU app. As no user accounts are needed for using the StudyU app, no identifying data are collected through the StudyU app. To link the data from the single-case experimental designs to further data collected in baseline questionnaires, participants enter the StudyU app using an invite code, which acts as a pseudonym. All data collected through the StudyU platform are stored on secure servers at the Hasso Plattner Institute in Germany and will be published openly through the StudyU Designer website [17] after study completion and after deletion of the invite codes to guarantee anonymized data. This will contribute to an open worldwide data repository, allow aggregation of single-case experiments testing similar interventions in future open science approaches, and inform the design of future trials.

### Design

#### Setting and Participants

The study will be conducted in a large privately owned general practice north of Brisbane, Australia. The practice serves a predominantly low socioeconomic patient demographic and has a commitment to practice-based research that improves patient care in their community. There were 5516 presentations for pain as the primary reason for attendance in 2020 to 2021 at the practice. The practice team has worked with us to design operational procedures to embed StudyU-enabled self-experiments in chronic disease and pain management consultation workflows and systems. We will recruit 4 GPs, 4 practice nurses, and up to 50 patients with a diagnosis of persistent pain, and we estimate that 30 patients will complete the study, an acceptable number for a feasibility study [18].

#### Inclusion Criteria

Participants will be eligible for inclusion if they meet the following criteria: (1) aged >18 years; (2) currently experiencing clinically significant persistent pain for 3 months or longer, most days per week (average pain severity in last week of at least 3-4/10); and (3) on stable doses of regular pain medication (including medicinal cannabis) for ≥4 weeks prior or not currently taking pain medication.

A subgroup of participants will be recruited with an additional inclusion criterion of “persistent neck and/or back pain following a road traffic crash at least 3 months prior to recruitment.”

#### Exclusion Criteria

Participants will be excluded if they meet any of the following criteria: (1) acute mental health disorder or suicidal; (2) unable to use digital health apps due to impairments in cognition, vision, or dexterity; (3) non-English speaking; (4) no access to a smartphone or the internet; or (5) recent (within the last 4 weeks) or planned (within the next 3 months) changes to current pain management interventions, including surgery.

#### Research Plan

**Figure 1:**
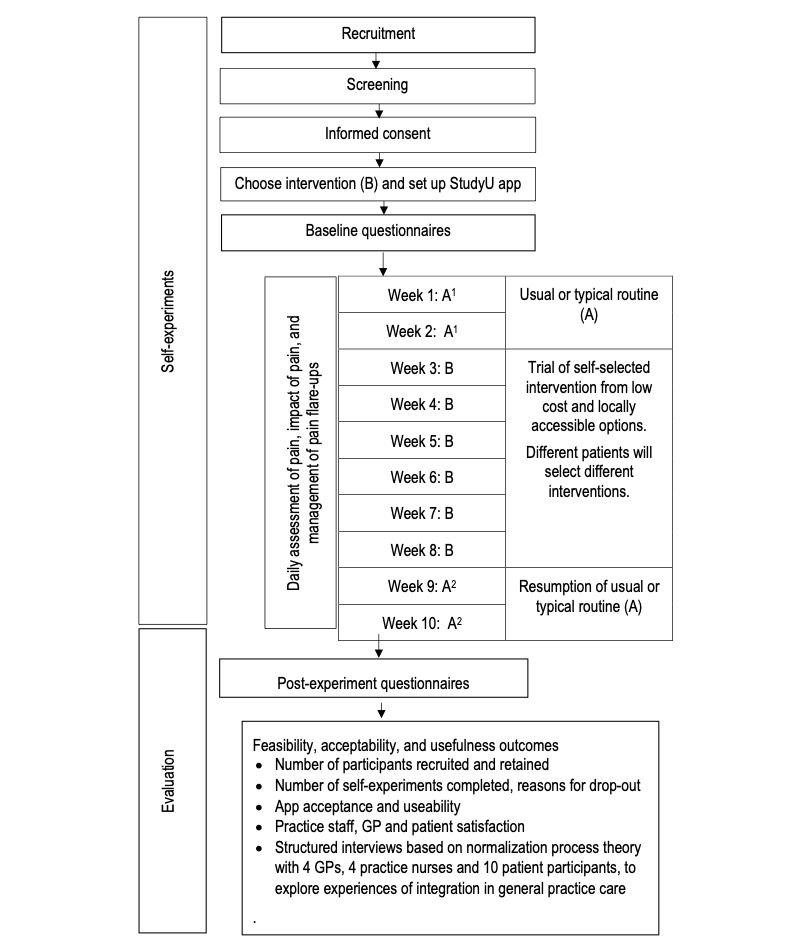
Overview of participant flow and study design. GP: general practitioner.

A patient flyer about the study and a 1-page explanatory information sheet will be displayed in practice waiting areas and consulting rooms. Study GPs and/or practice nurses will provide further information to patients who express an interest in participating, screen them for study eligibility, invite eligible patients to provide written informed consent to participate, assist patients to select a suitable nonpharmacological intervention, and complete study referral information. Refer to Figure 1 for an overview of participant flow and study design. A steering committee will oversee and advise the operational research team (the project manager [JN], the research assistant [SF], and the principal investigator [NS]).

#### Intervention

A research assistant embedded within the participating general practice will prepare and regularly update a list of low-cost activities generally recommended for the management of persistent pain and are locally available at low or no cost to practice patients. The activities are reviewed by a pain clinician to ensure they are generally appropriate. The list includes mindfulness practice, online pain management modules, low-impact exercise such as chair yoga or tai chi, walking programs (self-managed or group), and personalized or small group exercise programs. The patient selects an activity from this list with the approval of their GP. On receipt of the study referral, which is generated and autopopulated from existing practice software, the research assistant designs a personalized self-experiment for each patient using the StudyU Designer and forwards an invitation code to the patient for them to enter into the StudyU app to commence their personalized self-experiment.

Patients are assisted by the study team or practice nurses to download the StudyU app and enter their personalized invitation code. The patient’s self-experiment will continue for a total of 10 weeks, using an A1-B-A2 withdrawal/reversal single-case experimental design, where phase A1 is the patient’s usual or typical routine, phase B is 6 weeks of undertaking the intervention activity, and phase A2 is resumption of the usual routine (except for patients who wish to continue the intervention activity).

After the patient has completed their study, they will be encouraged to book a consultation with their GP to discuss the study results. This consultation will include shared decision-making about whether to continue the activity. Patients may also choose to conduct a second self-experiment with another activity.

### Outcome Measures

The primary clinical outcome measured daily in the app-enabled self-experiments is pain interference, measured using the modified Brief Pain Inventory 7-item interference subscale [19]. A change of 1 point in the average score across the 7 items is defined as clinically significant [20]. The secondary clinical outcomes are pain intensity, using a visual analog scale [21], and the number of additional treatments for pain flare-ups. All clinical outcomes are measured in the StudyU app.

In addition to these clinical outcomes, patients will also complete validated measures at baseline and 4 weeks following their self-experiment of self-efficacy, mental health, self-reported health status, health service use, and quality of life (refer to Table 1 for the full list of instruments used).

**Table 1:** Summary of outcome measures.

Outcome type	Primary outcomes	Secondary outcomes and assessments
Clinical outcome measures	Primary clinical outcome (measured in StudyU app): pain interference (modified Brief Pain Inventory interference subscale) [19]	Secondary clinical outcomes (measured in StudyU app): pain severity [21] and use of additional treatments for pain flare-ups Measured pre– and post–self-experiment: pain self-efficacy (Pain self-efficacy Questionnaire) [22] Depression, anxiety, and stress (Depression Anxiety Stress Scale) [23] Health-related quality of life (Kemp QOL^a^ scale) [24] Self-reported health status Self-reported health care presentations in the previous 3 mo In patients with persistent neck or back pain following a road traffic crash: Neck Disability Index [25] and Oswestry Disability Index [26]
Evaluation of StudyU app	Surveys assessing technology usability, usefulness, and acceptance (1-question System Usability Scale) [27] Acceptability of StudyU (unified theory of acceptance and use of technology) [28]	Semistructured interviews with participants during follow-up Document participant experience with and feedback on the app Assess any perceived impact on their pain self-management Assess concordance of postexperiment behavior with StudyU results
Evaluation of self-experiments	Feasibility and acceptability outcomes Number of participants recruited and retained Baseline/postintervention response rates Number of self-experiments completed, and reasons for dropout Practice staff, GP^b^, and patient satisfaction with self-experiments	Process evaluation Structured interviews using Normalization Process Theory [29] 4 GPs, 4 practice nurses, and 10 patient participants to examine implementation barriers and enablers.

^a^QOL: quality of life.

^b^GP: general practitioner.

We will examine the feasibility and acceptability of integrating digitally enabled self-experiments over the 12 to 18 months study duration using a nested process evaluation and structured individual interviews informed by Normalization Process Theory [29], with study GPs and practice nurses following study completion. Patients will complete measures of technology acceptance and usability (Table 1) and participate in structured interviews after their self-experiments to explore their experiences. Feasibility outcome measures include the number of participants recruited and retained and the self-experiment completion rate. All management decisions and reasons for dropout will be recorded by the research team. Outcome measures are summarized in Table 1.

### Statistical Analyses

Both individual-level and population-level analyses will be conducted. To answer the primary research questions regarding feasibility and usability, we will analyze data collected across participants and provide descriptive statistics that summarize usability, usefulness, acceptability, and feasibility outcomes. To assess the effectiveness of clinical interventions at the individual level (ie, causal effects), we aim to test whether the primary and secondary clinical outcomes would differ between phases when the intervention was applied and when it was not applied (standard routine), for each individual separately. For this, we will perform *t* tests comparing average daily scores of primary and secondary clinical outcomes between usual routine and intervention phases, as well as Bayesian linear mixed models to calculate the posterior probability that the intervention is effective at the individual level. We will consider 2 definitions of treatment responders: having an estimated posterior probability higher than 80% of a reduction by at least 1 unit in pain interference (ie, in the average of the 7 items in the modified Brief Pain Inventory 7-item subscale [20]) and of any reduction in pain interference. It may be possible to perform these analyses at an aggregate level across patients if multiple participants test the same intervention. In all analyses, the primary analysis will assume that data are missing completely at random and will perform analyses of the complete-case data (for each individual separately) without imputation. Sensitivity analyses will test the robustness of the results after multiple imputation of missing data. For the clinical reports, we will report the results both from *t* tests and Bayesian linear mixed models. All analyses will be performed using the statistical software R (R Foundation for Statistical Computing). Structured interviews will be transcribed and analyzed thematically using template analysis [30].

As our primary aim is to evaluate the feasibility of embedding self-experiments in general practice, we did not perform statistical sample size calculations for study planning. Regarding the self-experiments, our primary aims are to test the feasibility of reporting the results to clinicians and patients. Regarding the reported results of the trials, the effectiveness of interventions will be tested at the individual level. For these analyses, 70 measurements (1 daily measurement for 10 weeks) will be available for each patient if there are no missing data. For a patient with 20% missing data, a 2-sided 2-sample *t* test at a significance level of .05 would have 80% power to identify treatment effects with an effect size of Cohen *d* of at least 0.53.

## Results

**Figure 2:**
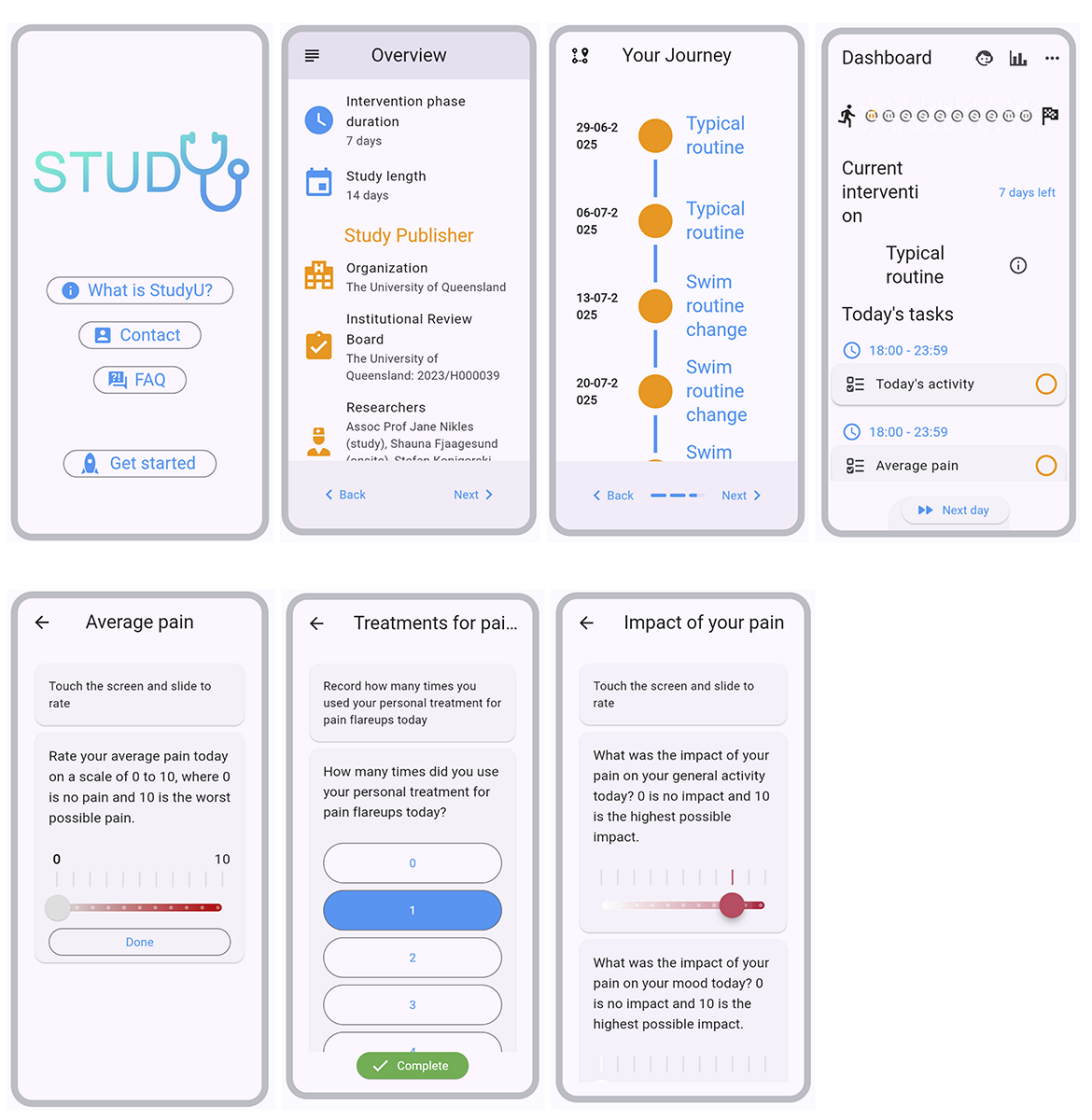
Illustrative screenshots of the StudyU App.

**Figure 3:**
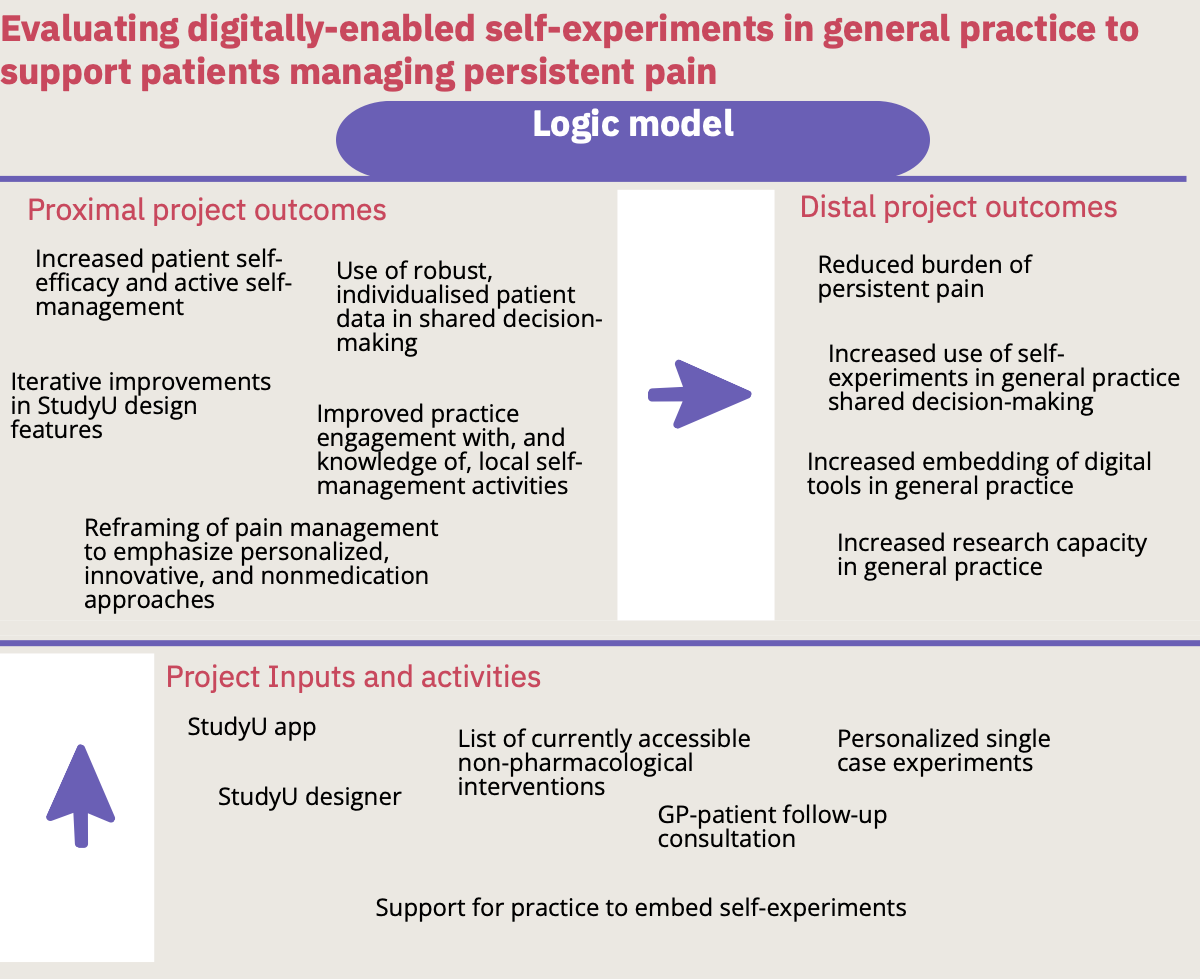
Project logic model. GP: general practitioner; SCED: single case experimental design.

The research was funded in September 2023 by the Medibank Better Health Foundation–Royal Australian College of General Practitioners Foundation 2023 and Motor Accident Insurance Commission–Royal Australian College of General Practitioners Foundation 2023 grants (MBHF23-01 MAIC23). Full funding and other agreements were completed by December 2024. Practice protocols have been developed, and practice staff have received training in the StudyU app and patient recruitment. Patient recruitment is scheduled to start in January 2025. Results are expected by December 2025. Refer to Figure 2 for screenshots of the StudyU app and Figure 3 for the project logic model, including inputs, proximal outcomes, and distal outcomes.

## Discussion

### Anticipated Findings

In addition to the clinical benefits for individual patients of identifying effective (or ineffective) treatments, the study findings will cast light on the feasibility, acceptability, and usefulness of embedding digitally enabled single-case experiments as a new tool for GPs to support patients with persistent pain.

### Strengths

Single-case experimental designs, despite some limitations, provide more robust evidence of intervention effectiveness than the more common “try it and see” approaches in general practice [31]. Digitally enabled single-case experiments are a novel approach in general practice. Patients are able to choose an intervention that is personally attractive, and we anticipate that use of the StudyU app will facilitate adherence to the planned intervention through the daily prompts and patient interest in establishing its effectiveness, potentially enhancing patient self-efficacy in relation to the self-management of their pain. We have addressed several known barriers to integration into general practice care by embedding the self-experiments in existing practice workflows and systems and facilitating patient referrals to locally accessible and affordable community-based services and activities.

### Limitations

The relatively short duration of the self-experiments limits our ability to detect any intervention effect with a delay in onset longer than 3 to 4 weeks or assess longer-term effects (beyond 4 weeks after experiment completion) of the intervention. The ABA withdrawal/reversal single-baseline design does not include within-case replication, assessment of baseline stability, blinding, randomization, and does not include additional blocks that may prevent wash-in (delayed onset) or wash-out (carryover) effects of the interventions. We considered incorporating some of these design features to increase the validity of self-experiment results [32] but considered that further increasing the length and complexity of the self-experiments was likely to reduce patient interest and engagement in our pilot feasibility trial. However, we exclude patients with recent or intercurrent changes in other pain management interventions or with cancer-related pain (which may be progressive). Limitations and caveats can be discussed during the follow-up GP-patient consultation, and future studies could optimize the length and design of trials for particular interventions. We are also testing StudyU-enabled self-experiments in a relatively small study in a single practice that is already research friendly and has provided funds to support the initial pilot; this limits the generalizability of our feasibility results to other practices that may have additional barriers to successfully integrating StudyU into clinical care. Patients self-select into the study; non-English speakers are excluded; and we have not provided devices, data, or internet access as strategies to enhance engagement, reducing patient diversity and representativeness. The restrictions are important for interpreting generalizations of the study results; in particular, it would not be possible to conclude that digitally enabled self-experiments are appropriate for all patients with persistent pain. The self-selection of participants interested in performing the trials is a feature rather than a limitation, as we believe that studying the effectiveness in this particular group is of interest. We have not attempted to standardize or check the fidelity of selected interventions or self-reported behaviors, which may make it more difficult to aggregate these single-case experiments in future studies.

### Implications for Practice

In addition to the implications for patients with persistent pain, findings may also contribute to a model for integrating other technologies and single-case experimental designs into the general practice care of patients with other chronic conditions.

## Abbreviations

GP: general practitioner
